# Performance of Biodegradable Active Packaging in the Preservation of Fresh-Cut Fruits: A Systematic Review

**DOI:** 10.3390/polym16243518

**Published:** 2024-12-18

**Authors:** Oscar T. Rodriguez, Manuel F. Valero, José A. Gómez-Tejedor, Luis Diaz

**Affiliations:** 1 Energy, Materials and Environment Group GEMA, School of Engineering, Universidad de La Sabana, Campus del Puente del Común, Km. 7, Autopista Norte de Bogotá, Chía 140013, Colombia; oscarrodga@unisabana.edu.co (O.T.R.); manuelvv@unisabana.edu.co (M.F.V.); 2 Centre for Biomaterials and Tissue Engineering, Universitat Politècnica de València, 46022 Valencia, Spain; jogomez@fis.upv.es; 3 Biomedical Research Networking Centre in Bioengineering, Biomaterials and Nanomedicine (CIBER-BBN), 46022 Valencia, Spain; 4 Bioprospecting Research Group, School of Engineering, Universidad de La Sabana, Campus del Puente del Común, Km. 7, Autopista Norte de Bogotá, Chía 140013, Colombia

**Keywords:** fresh-cut fruits, active packaging, shelf life, biodegradable, films

## Abstract

Fresh-cutting fruits is a common practice in markets and households, but their short shelf life is a challenge. Active packaging is a prominent strategy for extending food shelf life. A systematic review was conducted following the PRISMA guidelines to explore the performance and materials used in biodegradable active packaging for fresh-cut fruits. Sixteen studies were included from a search performed in July 2024 on Scopus and Web of Science databases. Only research articles in English on biodegradable active films tested on cut fruits were selected. Polysaccharides were the most employed polymer in film matrices (87.5%). Antioxidant and anti-browning activities were the active film properties that were most developed (62.5%), while plant extracts and essential oils were the most employed active agents (56.3%), and fresh-cut apples were the most commonly tested fruit (56.3%). Appropriate antioxidant, antibacterial, and barrier properties for fresh-cut fruit packaging were determined. Furthermore, there is a wide range of experimental designs to evaluate shelf-life improvements. In each case, shelf life was successfully extended. The findings show that different storage conditions, fruits, and material configurations can lead to different shelf-life extension performances. Thus, biodegradable active packaging for fresh-cut fruits has a strong potential for growth in innovative, sustainable, and functional ways.

## 1. Introduction

Fresh-cut fruits are commonly sold due to their ease of handling and convenient accessibility for households and consumers, who are interested in ready-to-eat foods [1,2]. However, preserving these products is a challenge that households and markets must address. Nutrient loss and microbial spoilage are common in fresh fruits following peeling and cutting [3]. Processing, such as cutting, triggers an increase in respiration rates and biochemical responses that leads to enzymatic browning [4]. Fresh-cutting also increases susceptibility to deterioration, microbial spoilage, and oxidation due to tissue damage and the loss of the natural barrier provided by the peel [1]. These factors contribute to a reduction in the fruit’s shelf life, generally of between 1 and 3 days, and quality loss [3,4,5]. A short shelf life is one of the main causes of food loss and waste across the world and is closely related to physiological, environmental, and storage conditions.

Food packaging is a prominent solution to this problem as it provides barrier protection against external factors such as moisture, microbes, and physical damage to extend food shelf life [6]. Researchers have focused on active packaging development, which is a food protection and shelf-life extension strategy. Active packaging interacts with the inner atmosphere by releasing active components (antioxidants or antimicrobials) or scavenging undesirable gases (oxygen or ethylene) to prevent food spoilage and extend shelf life [7].

The environmental implications of petroleum-based plastics are considerable, largely due to their processability, the emissions linked to this processability, and their non-degradability [8,9]. Consequently, the development of biodegradable or biobased materials is encouraged [9,10]. Biobased materials are derived from renewable resources, while biodegradable materials degrade via biological action [11], with renewability, non-toxicity, abundance, and degradability being some of the advantages of biobased plastics [12,13]. Polysaccharides, proteins, and lipids are food packaging material alternatives [12].

As there are so many raw materials that can be combined in many different ways, different properties and performances have been observed, and it is difficult to compare these properties in general terms. The performance of novel packaging films is defined by their barrier, thermal, mechanical, flexibility, and active properties, which are crucial for their effective application, the preservation of quality, and the extension of shelf life [14]. Shelf-life determination is an important factor to evaluate active packaging. In many cases, studies on active packaging development have measured shelf life to determine potential applications of active packaging materials. The physiological properties (weight loss, firmness, color, acidity, and soluble solids) and decay spoilage (bacterial or mold counts) of the tested foods are some of the other aspects evaluated.

Since shelf-life testing is highly relevant and plays a crucial role in determining the success of packaging materials, the results reported in the active packaging literature should be explored, contributing to the diversification and development of new and effective active packaging materials. Thus, the objective of this review is to study the performance of biodegradable active packaging films using shelf-life tests of fresh-cut fruits in publications from the last 10 years.

## 2. Materials and Methods

### 2.1. Review Design and Eligibility Criteria

This systematic review followed the PRISMA guidelines [15]. The methodology consisted of searching for, identifying, and analyzing studies from the active packaging literature related to biodegradable films tested on fresh-cut fruits. The information sources were the scientific databases Scopus and Web of Science. Studies were selected using the following inclusion criteria: (1) only research articles, (2) publications from 2013 to 2024, (3) active packaging material, and (4) biodegradable materials. The following exclusion criteria were used: (1) reviews and book chapters; (2) publications in languages other than English; (3) active packaging that is not in the form of a film; (4) active packaging under modified atmosphere conditions; and 5. studies that evaluated the shelf life of foods other than cut fruits.

### 2.2. Information Sources and Search Strategy

The reviewed information was obtained from the scientific databases of Scopus and Web of Science. The search was performed in July 2024. The search strategy was developed using a keyword and Boolean query. The query “(All Fields) [food packaging films AND fruit* AND biodegradable]” was used in Web of Science with the filters, (Publication years) [2013–2024] and (Document types) [Article], and the query “(All fields) [“food packaging films” AND “cut fruit*” AND biodegradable]” was used in Scopus with the filter (Years) [2013–2024].

### 2.3. Study Selection Process

The web application of the reference manager Rayyan was used for article management. First, the title and abstract of each study were reviewed by three independent researchers (OTR, MFV and LED). Subsequently, the three researchers evaluated and classified the articles as either approved or rejected. In cases of discrepancies (where two researchers approved, and one rejected), the articles were collaboratively reviewed to reach a definitive decision on their approval or rejection. The approved studies were read in full, and those that did not meet the inclusion criteria were filtered out again.

### 2.4. Data Collection

To collect data, a database form was developed and used to summarize the following information: article title, corresponding author names and countries, year of publication, journal name, raw materials for film synthesis, active substances, evaluated activities, prepared fruit samples, shelf-life evaluation, and film characterization.

## 3. Results and Discussion

### 3.1. Main Results

A total of 577 articles were identified during the search stage, including 261 from Scopus and 316 from Web of Science. From these, 10 articles were removed as duplicates. After screening the remaining articles, 356 were excluded. The main reasons for exclusion were as follows: a lack of shelf-life tests on methodology, food other than fruits, review articles, overviews, and chapters.

**Figure 1:**
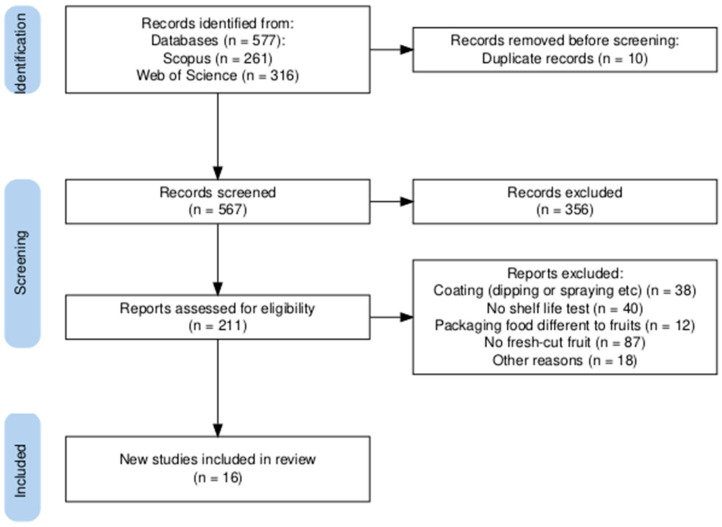
PRISMA flow diagram [22].

Of the 211 potentially eligible articles, 195 did not meet the inclusion criteria as they focused on shelf-life tests for packaged uncut fruits, for example, bananas [16], strawberries [17], grapes [18], and mangoes [19], among others, or tests on fruit samples coated by immersion [20] or spraying [21] instead of film-wrapped samples. Thus, 16 studies were included for data collection (Figure 1).

**Figure 2:**
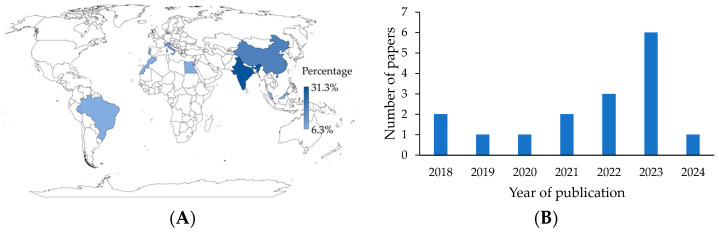
(**A**) Geographical distribution of research. (**B**) Publication distribution over the years.

The first consideration is the location of the study, for which the country to which the corresponding author was affiliated was selected. In general, nine countries contributed publications to the active packaging literature. It is worth noting that India (31.3%) and China (18.8%) are advanced in this research field (Figure 2A). This may be due to the growing interest in using eco-friendly materials for food packaging and in reducing food loss and waste. These concerns may be particularly relevant in highly industrialized, populated, and biodiverse countries, where governments, entities, and organizations recognize that eco-friendly packaging is of utmost importance in the future [23]. An increase in publications was also noted. More than half (81.3%) of the articles were published in the last five years (Figure 2B). In addition, 18.8% of the studies were from Europe, 12.5% from Africa, and 6.3% from America.

### 3.2. Main Polymers for Active Packaging

Several concerns have been raised about the environmental impact of using petroleum-based plastics in food packaging, as they are single-use and contribute significantly to waste [24]. This highlights the advantages of biobased and biodegradable polymers over petroleum-based polymers. In fact, the environmentally friendly end-of-life options of bioplastics and the fact they come from renewable sources make them a sustainable alternative. Furthermore, naturally occurring polymers offer additional advantages due to their non-toxicity, making them a safe and reliable choice for food contact applications [25].

For ASTM, biodegradable plastics refers to materials that break down into water, carbon dioxide, or biomass through natural processes and the action of microorganisms like bacteria, fungi, and algae within a reasonable period of time [26]. In general, biodegradable materials are those that degrade into simpler non-toxic substances [27,28]. The European Union standard UNE-EN 13432 stipulates that biodegradable materials must decompose to at least 10% of their original mass within 3 months under industrial composting conditions [29]. These definitions offer a comprehensive view to evaluate novel biodegradable active packaging.

There are several strategies for biodegradable plastic film fabrication; the use of residual biological mass has gained attention in this regard. Some of the most common and important polymers employed as raw materials are polysaccharides and proteins [30]. Additionally, the substitution of non-biodegradable petroleum-based plastics is being explored through the use of synthetic biodegradable polymers such as polyvinyl alcohol (PVA), poly (butylene adipate-co-terephthalate) (PBAT), polycaprolactone (PCL), polylactic acid (PLA), and polyhydroxyalkanoates (PHAs) [31].

Polysaccharides such as starch, cellulose, pectin, chitosan, and others are among the most employed due to their abundance (some are by-products of the agricultural industry), biocompatibility, and biodegradability [32,33,34,35]. Of the articles included, 87.5% employed polysaccharides to fabricate film packaging, followed by synthetic biodegradable polymers and then proteins to fabricate. Composites of different polysaccharides, polysaccharides with proteins, or polysaccharides with aliphatic polyesters were commonly used to fabricate film matrices with key capabilities. Therefore, composites were used as the main component of active packaging films in most of the studies.

Chitosan and cellulose derivatives are among the most used polysaccharides, each appearing as raw materials in 31.3% of the studies. Chitosan is a frequently used alternative as it is highly abundant and has antimicrobial activity. This activity is due to the deterioration of microbe cell walls via negative–positive reactions with the polymer [36]. The crystalline structure of cellulose results in materials with a high stiffness, durability, and thermal stability [36]. Starch closely follows, as it was used in 12.5% of studies. Starch is odorless, colorless, and chemically inert and exhibits film-forming abilities, which are some of the reasons for its wide utilization [37]. These polymers are low-cost and suitable carriers for different active substances [36].

Synthetic biodegradable polymers were the second most employed materials in the chosen investigations following polysaccharides. Synthetic biodegradable polymers have been found to be non-toxic, biocompatible, and to exhibit a good processability [38]. These classes of raw polymer material, mainly blended with natural polymers, were considered in 37.5% of the studies.

Some aspects where biodegradable packaging falls short compared to petroleum-based alternatives are its thermal and mechanical properties, which may limit its performance in certain applications. As shown in this review, blending polysaccharides with synthetic polymers is a common strategy to improve mechanical properties such as tensile strength, elongation at break, and Young’s modulus [39]; improve processability, such as spinnability in the case of polysaccharides [39]; and reduce manufacturing costs in the case of synthetic polymers [40].

Protein gelatin has been an increasingly prevalent raw material in many applications, such as in the food and pharmaceutical industries. It is widely used because of its film-forming capacity, transparency, elasticity, and protective properties against light and temperature [41]. Nearly all vegetable proteins, such as wheat gluten, corn zein, and soy protein, among others, have found their way into food packaging applications [42]. In total, 18.8% of the articles employed these proteins; they were the least commonly used raw materials.

Except for chitosan, other polymers used in film packaging act as a passive barrier. Thus, to promote shelf life, bioactive substances must be incorporated into polymer matrices.

### 3.3. Active Principles

Active packaging for fresh-cut fruits typically provides antioxidant, anti-browning, or antimicrobial effects. Antioxidant activity is achieved by the removal of free radicals accepted by scavenger molecules with radical scavenging capabilities, such as polyphenols, flavonoids, and terpenes [43,44]. This is the main activity reported in active packaging for fresh-cut fruits, reported in 62.5% of the studies.

Anti-browning activity is a major advantage for quality maintenance in fresh-cut products. Some ways to prevent browning are to inhibit the enzymatic action of polyphenol oxidase (PPO) by creating an oxygen barrier or using active packaging with a scavenging capacity [45]. A total of 62.5% of studies characterized this activity as a further potential capacity of films.

Antibacterial activity has important repercussions, mainly related to quality conservation and food security. Fresh-cut fruits provide a favorable environment for moisture loss, browning, and microbial growth due to the presence of nutrients and surface area exposure [46,47,48]. Given consumer concerns, active packaging may offer a viable alternative to chemical preservative-based additives [49]. A total of 50% of the studies promoted and evaluated this activity. Two studies focused on inhibiting fungi, while the others focused on inhibiting bacterial strains.

From this review, it seems that the substances with a higher potential are nisin [30], high-phenol- and flavonoid-content extracts like grapefruit and lemon peels [50], watermelon rind [51], and noni leaf extracts [52]. Among the active polymers, chitosan had the most prominent antimicrobial effects.

Further, research on active packaging with both antioxidant and antimicrobial activities has the potential to be expanded; only 31.3% of the studies studied the presence of these two properties at once. Similarly, a wide variety of natural substances have been proposed. Plant extracts and essential oils are the most common source of bioactive components. Examples of sources include shallot [53], Ficus [54] and lemon plant [50] extracts, as well as olive [55], cinnamon [12] and lavender [56] essential oils, which are used to introduce antioxidant and antibacterial properties. A mixture of phenolic components in plant extracts promotes antioxidant and antibacterial properties. Nonetheless, their incorporation into polymer matrices has led to mechanical, physicochemical, and barrier modifications [57].

Plant extracts and essential oils are the main active agents employed in active packaging in 56.3% of studies. These are agents with a high biological activity and are principally composed of phenolic and flavonoid compounds, which promote antioxidant and antibacterial activity by acting as scavengers of free radicals and modifying cell membranes or bacterial DNA, thus inhibiting their growth [58].

### 3.4. Film Fabrication Procedures and Biodegradability Assessments

Three distinct manufacturing procedures have been employed for the production of films, namely electrospinning, extrusion, and casting, based on a polymer’s capacity to be electrospun, extruded, or cast, respectively. All of the aforementioned methods are typically initiated using polymer solution mixtures, comprising a combination of polymers and fillers. The solution casting method is the most prevalent due to its simplicity, cost-effectiveness, and suitability for active packaging based on thermosensitive molecules, as well as the fact that it can be used for large-scale production of biodegradable films [59].

As previously stated, most methodologies entail the incorporation of active substances prior to the formation of the polymeric matrix; these active substances are generally fillers. Consequently, experimental film design has been conceptualized as a means of assessing the impact of augmented quantities of active substances, such as fillers, on the characteristics and functionality of packaging films. For instance, according to the reviewed articles, an increase in the proportion of filler results in a deterioration of the film’s mechanical properties due to the occurrence of phase separation, failure points, or discontinuities in the polymer matrix [40,51,55].

Deposition via coating or immersion is a novel approach that has not yet been widely explored. In fact, only one article employed this, noting no significant changes in the mechanical performance [60].

To assess the biodegradability of packaging films, the reviewed articles exclusively employed the soil burial test, which simulates natural environmental conditions. In this method, the degradation of the samples is evaluated over time by measuring their weight loss. The results suggest that biodegradable active packaging films degraded easily; however, the active substances appeared to affect the degradation rate through their antioxidant, antimicrobial, or hydrophobic properties [52,54,61].

The protocols used in the reviewed articles do not explicitly specify which ISO, ASTM, or other standard testing methods were applied, and they differ slightly from one another. Future investigations would benefit from referencing standardized protocols and incorporating additional biodegradability methods, such as testing under marine conditions and composting, using standards using the standards mentioned by internationals organizations; ISO, ASTM, and UNE-EN, among others. These approaches would provide a more comprehensive evaluation of the environmental impact of biodegradable active packaging films.

### 3.5. Most Commonly Tested Fresh-Cut Fruits

Fresh-cut fruit has advantages in terms of ease of handling and manipulation; these products are ready to use and consume and are easier to store on shelves and in refrigerators, which is especially useful for bulky fruits like watermelon and melon. In this review, it was observed that eight types of fresh-cut fruits were tested under storage with active packaging. Of these, apples were the most studied, as they were the focus of 56.3% of the studies. The rest of the studies validated fresh-cut watermelons (two articles), bananas (one article), tomatoes (one article), kiwis (one article), melons (one article), mangoes (one article), strawberries (one article), durians (one article), and pomegranates (one article).

Despite the easy-to-handle nature of the cut fruit, fresh-cutting has a negative impact on fruit quality. Physiological, chemical, and sensory changes occur quickly, some of which are catalyzed by enzymes such as polyphenol oxidase [62]. Freshly cut apples become rapidly oxidized and browned due to the exposure of polyphenol oxidase to the environment and soften due to the hydrolysis of pectin [63]. Fresh-cut apples’ rate and propensity to oxidation and browning mean they are commonly chosen as the primary means of validating the performance of active packaging [64].

In general, the methods for preparing samples for shelf-life testing involve selecting fruits based on their healthy appearance, their size uniformity, their stage of ripeness, and the absence of physical and microbial damage. The fruits are then washed with distilled water and, in some cases, decontaminated with sodium hypochlorite to remove dirt and sanitize them. Further, fruits are peeled and cut into slices or cubes. Finally, the fruits are packed in the active films developed in the study and stored under defined temperature and humidity conditions. The test time ranges from 6 h to 14 days.

### 3.6. Performance of Active Packaging Films

Different biodegradable active packaging exhibits various performances. Each study has its own characteristic experimental design, requiring careful review and interpretation. Although the results are difficult to compare across investigations, general highlights are presented in Table 1.

### 3.7. Antioxidant Advantages

In all the studies, it can be observed that antioxidant packaging films have an advantage with regard to maintaining physical and sensory qualities. Oxidation processes affect the quality of perishable foods, which can be noticed by color, flavor, and texture deterioration [67]. In fresh-cut fruits, enzymatic browning and oxidation of substrates shorten their shelf life. One of the most common anti-browning agents are essential oils [68]. Consequently, researchers have shown interest in extracts enriched with flavonoids, phenols, alcohols, and other bioactive molecules, including essential oils from thyme [69], cinnamon [32], clove [70] and shallot [53], as well as extracts form watermelon [51] and grapefruit [50], among many others. Briefly, all studies have confirmed that antioxidant active packaging prolongs the shelf life of fresh-cut fruits. Generally, DPPH and ABTS assays have become the go-to tests for antioxidant capacity measurements for active packaging. This may be due to their sensitivity and production of quantifiable results.

### 3.8. Antibacterial Advantages

Microbial contamination often causes premature spoilage of food, compromising its quality and safety [71,72]. Bacterial contamination is the primary concern when it comes to food safety. *E. coli* and *S. aureus* are the most representative Gram-negative and Gram-positive bacterial pathogens, respectively, while *C. albicans* [52], *Fusarium* sp. [73], and *B. cinerea* [74] are the most common yeast and fungi. They easily contaminate food and thus were tested in almost all studies using antimicrobial assays to evaluate active packaging materials. Controlling and mitigating these pathogens play a crucial role in extending the shelf life of fruits, and packaging provides a fundamental solution to this issue.

The rate of microbial growth is slowed using packaging. The reviewed articles suggest that unpackaged fresh-cut fruits exhibit the highest rate of microbial spoilage. Biodegradable packaging can improve properties such as water transmission and control, leading to reduced moisture contents and less favorable conditions for microbial growth, as was the case with polysaccharide-based polymers [30]. However, antibacterial active packaging designs were even more effective in reducing spoilage, as demonstrated in the case of chitosan-based materials, which introduced synergistic barrier and active properties [51,60].

The results demonstrate that active substances and polymers can promote bacterial inhibition in active packaging. Antimicrobial active packaging is effective in controlling microbial growth (bacteria and fungi) [72]. Thus, antimicrobial packaging can extend the shelf life of fresh-cut fruits and act as a food security strategy.

### 3.9. Positive Characteristics of Active Packaging Films

The encapsulation of bioactive compounds has emerged as a prominent strategy to overcome the drawbacks of active compounds, including their instability, volatility, oxidation, or interference with the sensory attributes of packaged food [12,50,66,75]. Encapsulation strategies include cyclodextrin inclusion complexes [75], nanoliposomes [66], maltodextrin [50] and polydopamine capsules [12]. The results highlight the enhanced stability, improved release performance, and odor masking ability of these methods. Thus, encapsulation may play a significant role in the next generation of active packaging.

Moreover, the sensory qualities of fresh-cut fruits, which are crucial for evaluating active packaging, indicate promising results. Color was the primary studied quality characteristic, and as previously stated, anti-browning and antioxidant packaging are more effective at maintaining it. A study that included a sensory evaluation reported that fresh-cut fruit stored in biodegradable active packaging scored higher with regard to sensory characteristics compared to those stored in traditional petroleum-based polyethylene (PE) packaging. The superior performance of the active packaging, attributed to its enhanced barrier and active properties, resulted in an extended fruit shelf life and improvements in appearance, texture, color, aroma, and taste [51].

In terms of extending the shelf life of fresh-cut fruits, biodegradable active packaging surpasses traditional methods by continuously mitigating food spoilage, often by using natural, food-grade components that are safe and sustainable, while traditional packaging acts as a passive barrier that does not prevent browning, oxidation, or microbial growth actively. Biodegradable active packaging has proven to be superior with regard to maintaining appearance [51] and lowering microbial spoilage [30] during fresh-cut fruit storage in comparison with PE.

Furthermore, there is a necessity for continued advancement in the development of biodegradable packaging films. While active properties may serve as a support, it is essential that mechanical, barrier, and thermal properties be subjected to rigorous analysis to ensure that these developments are competitive against commercial polymers. UV light barrier films are remarkable designs. Phenol functional groups provide favorable sites for ultraviolet (UV) absorbance [50]. Active packaging incorporating grapefruit and lemon extracts [50] or watermelon rind extract [51] functions effectively as a UV shield. UV protection is desirable to mitigate appearance deterioration and nutrient degradation.

Additionally, water loss from the fruit and moisture in the packaging may be regulated by designing films with an appropriate hydrophobicity, which improves the water resistance, decreases the solubility, and optimizes the water vapor transmission rate. The water transmission rate (WVTR) is a prominent characteristic which may be controlled by designing polymer matrices and fillers with an appropriate number of hydrophilic groups (OH, NH) as observed in [54]. Usually, a low water vapor transmission rate is desirable to prevent excessive moisture loss. However, for high-respiration fruits, a moderate transmission rate is necessary to maintain a balanced humidity level and avoid condensation and microbial invasion [39,64]. Multilayered films are noteworthy for their ability to regulate moisture levels in fresh-cut fruit. As reported in one reviewed study, the inner layer, with its combined hydrophilic–hydrophobic properties, regulates internal moisture, while the outer layer acts as a barrier against external moisture due to its hydrophobic nature [39]. In this case, this approach appears to be the optimal novel method for active packaging of fruits. It is versatile because it can be used for both high- and low-water-release fresh-cut fruits.

All the factors mentioned above—UV barriers, active mechanisms, and moisture permeability and control—have been shown to significantly influence the freshness of fresh-cut fruits. Moreover, in terms of nutrient retention, the reviewed articles conclude that the anti-browning and antioxidant properties of the packaging film provide outstanding defense against the loss of polyphenols in fresh-cut fruits [52]. Additionally, a low oxygen permeability, which reduces the available oxygen in contact with the fresh-cut fruit, is desirable to inhibit nutrient loss caused by oxidation reactions [51,53]. Moreover, vitamin C, being water-soluble, is susceptible to loss through water release. Therefore, it is best preserved when the packaging features a low water vapor transmission rate, which effectively minimizes moisture loss to the external environment [52]. Thus, the environment created by active packaging has significant beneficial effects beyond shelf-life extension, as it helps to preserve essential nutrients and maintain the sensory quality of the product.

### 3.10. Limitations of Active Packaging

In contrast, many compositions suffer from the low compatibility between the polymer matrix and fillers. The most common negative effects are presented in terms of mechanical performance indicators, of which the elongation at break is the most negatively affected property. Failure points and decreased mobility are the main causes of a lack of flexibility. The properties of polymers can be enhanced through the judicious selection of an appropriate active material, the development of composites, and the utilization of crosslinking agents and plasticizers.

The results of all studies indicate that fruit shelf life was successfully extended. Nevertheless, the lack of a standardized shelf-life testing methodology represents a significant concern. Future research must consider the type of fruit utilized, with conditions that can be standardized. Apples appear to be the optimal fruit for shelf-life experiments, as they are susceptible to browning and microbial spoilage, which can be quantified to some extent.

Another limitation of the selected fresh-cut fruit packaging studies is the inconsistency in storage conditions. It is essential that the relative humidity, temperature, and pH levels remain consistent between articles to mitigate the impact of external factors. It is imperative that only active packaging is the source of the observed effect. This approach allows for a comparison of active packaging designs.

Similarly, the diversity in packaging formats, including trays wrapped in the films, containers with film lids, bags, and direct wrapping, may influence the shelf-life outcomes. For example, for one type of application, containerized storage may be beneficial, whereas for another, direct wrapping may be preferable. However, this situation is a consequence of the lack of staging in fruit selection and/or applications.

A potentially good consequence of active packaging films is that the active molecules migrate to packaged food. Almost all studies indicate migration of components, but only two of them fully characterized the release kinetics. This migration may further enhance the food preservation [66]. However, it is mandatory to ensure consumers’ safety. Likewise, materials for active packaging films must be supported by entities like the Food and Drug Administration (FDA) or the Food and Agriculture Organization of the United Nations (FAO).

## 4. Conclusions

We have compiled a wide range of techniques and raw materials used for producing biodegradable active packaging for fresh-cut fruits, all of which can serve as a reference for developing new solutions or improving existing ones. Future research can leverage the findings reviewed here by exploring the application of specific polymeric matrices under standardized conditions, using the precedents set in these studies as a foundation.

According to our findings, the use of polysaccharides in active packaging is common, with chitosan being the most prominent polymer material. On the other hand, natural extracts are also potential sources of antioxidant and microbial activity. An appropriate combination of the polymer and active compound is one that contains two raw materials: a polysaccharide such as chitosan and a natural extract. These materials lead to favorable economic, biocompatible, and biodegradable properties.

Both antioxidant and antibacterial activities lead to considerable advantages. Therefore, combining both activities is a promising strategy. Studies on cut fruit have shown a significant decrease in enzymatic oxidation and microbial proliferation as a result of antioxidant and antibacterial active packaging. In general, active packaging extends the shelf life by between two and five days compared to passive packaging.

The active packaging literature has become increasingly diverse; no studies with common configurations or experimental designs have been reported, and many raw materials, active substances, and experimental designs have been employed. The lack of standardization makes it difficult to perform meta-analyses to determine the best configurations. For this reason, there is still a long way to go in terms of research on packaging with active materials for fresh-cut fruits. In this article, we highlight some properties of active packaging for fresh-cut fruits.

## Figures and Tables

**Table 1:** Main results of the studies on shelf-life extension included in the systematic review.

Polymer Matrix	Filler Employed	Film Manufacturing Method	Fresh-Cut Fruit	Main Result	Advantages	Disadvantages	Reference
**Category: Antioxidant and anti-browning active packaging**							
Polybutylene succinate (PBS)	Extra virgin olive oil (PBS/EVO 1, 2, 3%wt) or coconut oil (PBS/CO 1, 2, 3%wt)	The polymer–filler mixture was extruded into pellets and then blown into a film.	Apples and kiwi halves in direct contact with the developed films; stored for 12 days.	The shelf life of the fruits was monitored for 12 days. PBS (passive) and PBS/CO (active) films maintained the quality of fruits for nearly 3 days. PBS/EVO films (active) were shown to inhibit fungal spoilage in fruits for a further 5 days with a superior performance to that of PBS and PBS/CO films. Specially, PBS/3%EVO showed a better performance. Thus, PBS/EVO films increase the shelf life of fruits to nearly 8 days.	Water resistance is enhanced due to the increased hydrophobicity provided by oils. PBS/EVO films have an overall higher elongation at break (119.8% for 3% EVO).	Tensile strength (22.5 MPa, pristine PBS) is reduced by the addition of oils due to phase separation occurrence.	[55]
Sodium alginate (1% *w*/*v*)/carboxymethyl cellulose (1% *w*/*v*) Glycerol (0.02% *w*/*v*) CaCl_2_ and citric acid crosslinkers (0.2% *w*/*v*)	Shallot onion peel extract (0.2–0.5% *w*/*v*) Shallot onion stalk extract (0.2–0.5% *w*/*v*)	The film-forming solution was cast into films.	Apple slices wrapped in the developed films and stored in polypropylene trays for 12 days at 4 °C.	Sodium alginate/carboxymethyl cellulose films exhibited antioxidant activity provided by shallot onion extracts. Peel extracts at 0.5% in film provided the better overall performance, with nearly 92.28% and 78.82% radical scavenging abilities in the DPPH and ABTS assays, respectively. After 12 days of storage, apple browning was controlled.	Proper water resistance and barrier to vapor and oxygen due to adequate polyphenol and matrix interactions. The films demonstrated a biodegradability in soil of over 14% within 5 days.	Significant change in appearance, turning the films a red color.	[53]
Zein and chitosan Glycerol or PEG400 plasticizers	---	Chitosan or zein film-forming solutions were cast into films.	Apple cubes placed in containers covered with the developed films. Stored at 30 °C and 50% RH for 24 h.	Both chitosan and zein films showed antioxidant capabilities. However, the performance of the zein films (near 68%; DPPH assay) was better than that of the chitosan film (near 20%). After 24 h, the packaging performance in terms of fruit color was not significantly different between biopolymers and commercial PVC films. However, in terms of weight loss, PVC behaves better, at close to 3%, then zein films and chitosan films (close to 10% and 17%, respectively).	Acceptable mechanical properties of PEG-400 plasticizer formulations. Chitosan/50%PEG400 (TS > 50 MPa and EAB > 40%) had a superior overall performance.	Increased swelling, moisture content, and water-vapor permeability due to a lower hydrophobicity.	[64]
Chitosan (65%wt) Glycerol (15%wt)	Kaolinite clay (10%wt) and Ficus leaf extract (10%wt)	The film-forming solution was cast into films.	Apple pieces wrapped in the developed films and stored under ambient conditions for 24 h.	Ficus leaf extract can enhance the antioxidant activity of chitosan films from nearly 20% to 60% (free radical scavenging in DPPH assays) at a 10 mg/mL film equivalent. The active films show great performance in preserving the fruit for 24 h. The performance in terms of weight loss improved from nearly 27% in the pristine chitosan film to nearly 15% in the chitosan film with fillers. The color and phenolic content remained similar to those of fresh fruit.	Proper water resistance as well as barrier capacity due to the crystallinity and hydrophobicity of the material. UV light barrier protection gain. Thermal resistance (near 20% mass loss) up to 240 °C and superior mechanical properties (TS 21.08 MPa and EAB 33.35%). Films have developed biodegradability in soil.	The films turned green due to extract incorporation. Although kaolinite enhances biodegradability in soil (78% within one week), Ficus leaf extract reduces the degradation rate to 54% due to its antibacterial and antioxidant properties.	[54]
Poly(butylene adipate-co-terephthalate) (32–49%wt)/starch (32–49%wt) Glycerol (16%wt) plasticizer	Nanocellulose (0.45%wt), citric acid (0.4 and 0.8%wt), and annatto (0.8%wt)	The polymer–filler mixture was extruded into pellets and then extruded into films.	Mango pieces (2 cm × 2 cm^2^) were placed in a 15 cm × 20 cm film turned into a bag and stored at 5 °C, RH between 85 and 90% for 14 days.	The shelf-life performance of the formulations was comparable to that of commercial PVC films. The moisture content of the fruit remained near 90% for 7 days and near 80% on day 14. The absence of mold and yeast indicates the possible antimicrobial activity of annatto.	Slight improvement in the water-vapor barrier capability when fillers are incorporated.	Small reduction (<6.65 MPa) in the tensile strength in films with fillers due to some incompatibilities with the polymer matrix.	[40]
Gelatin (3% *w*/*v*) Glycerol plasticizer (0.6% *w*/*v*) Tween 20 emulsifier (0.018% *w*/*v*)	Durian leaf extract (0.2 and 0.5% *w*/*v*)	The film-forming solution was cast into films.	Durian portions were wrapped in films and stored at 4 °C for 4 weeks.	Durian leaf extract promoted antioxidant activity on gelatin films. The films loaded with 0.5% extract exhibited values of 0.44 and 3.445 mg/mL Trolox Equivalent per 100 mg of film in the DPPH and FRAP assays, respectively. Durian leaf extract modified the performance of the gelatin film, reducing fruit weight loss to 27% for the 0.2% loaded gelatin film and 33% for the 0.5% loaded gelatin film after 4 weeks. The commercial film (control) behaved slightly better at 17%. No significant differences were observed in the physical appearance of fruits packed in commercial or gelatin-leaf extract films.	Strong antioxidant capabilities.	The water-vapor barrier was not improved as expected. No other film characteristic was characterized.	[65]
Polyvinyl alcohol (PVA) (8% *w*/*v*)/ Kefiran (6% *w*/*v*) /Polycaprolactone (PCL) (15% *w*/*v*)	Flexirubin pigment (15%wt)	Double-layered film obtained via electrospinning. Inner layer: Kefiran:PVA (1:2). Outer layer: PCL.	Apple slices wrapped in the developed films and stored for 12 h. No other storage conditions were reported.	The incorporation of kefiran and flexirubin enhanced the antioxidant capacity; 41.03% ABTS scavenging can be achieved. Apple slices packed with this film can achieve a maximum weight loss of 4.5%.	No significant changes were observed in mechanical properties (TS 3.45 MPa and EAB 8.55%) and porosity with flexirubin incorporation.	High water transmission due to low hydrophobicity or high porosity.	[39]
**Category: Antibacterial active packaging**							
Chitosan	Vanillin nanoparticles	Chitosan solution was cast into a film and then immersed in the vanillin NP solution for 5 min.	Watermelons, melons, and strawberries cut in cylindrical plugs (2.5 cm diameter, 3 cm length), placed on square films and stored in polyethylene terephthalate containers for 9 days at 10 °C for watermelons and 12 days at 8 °C for melons and strawberries.	Antimicrobial effects against Gram-negative (*E. coli*) and Gram-positive (*S. aureus*) bacteria are based on chitosan activity. A significant reduction in CFU quantification (~6log) was achieved. No effect of vanillin nanoparticles was reported. Antibiofilm properties can be achieved only in *E. coli* when vanillin nanoparticles are incorporated into the chitosan polymer. With active packaging, microbial inhibition was significantly improved, remaining below the acceptability thresholds 4 days longer than the control (unpacked). Chitosan packaging yields approximately 4.5 bacterial and 2 mold–yeast counts (log CFU/g) on watermelon after 9 days of storage and near 7.7 bacterial and 6 mold–yeast counts (log CFU/g) on melons after 12 days. Furthermore, nearly 7 bacterial and 6.5 mold–yeast counts (log CFU/g) were detected in strawberries after 12 days. The incorporation of vanillin lowered these values to near 4 and 0 (watermelon), 5.5 and 4 (melon), and 5.7 and 5 (strawberries) bacterial and mold-yeast counts (log CFU/g), respectively.	Films are thermally resistant (11% mass loss) until 200 °C.	Mechanical properties and water-vapor permeability require further improvement (TS 24.24 MPa and EAB of 24.25%).	[60]
Agar (1.6% *w*/*v*)/carrageenan (0.2% *w*/*v*) Glycerol (0.6% *w*/*v*)	Nisin (0.2, 0.24, 0.28, 0.32 and 0.36% *w*/*v*)	The film-forming solution was cast into films.	Watermelon (2 × 2 cm squares) stored in plastic bowls covered with the developed films at 90% RH for 3 days at 4 °C, and 1 day at 20 °C.	The active film incorporates the antibacterial activity of nisin, as shown in in vitro assays. Inhibition circle widths increased from 0 mm at 0% nisin content to nearly 4 mm and nearly 2 mm against *S. aureus* and *L. monocytogenes*, respectively, in 0.36% nisin film. After 15 days of storage at 4 °C, the cut watermelon exhibited 2.4% weight loss. Bacterial spoilage can be slowed down for a few days. Quality parameters such as soluble solids, hardness, titratable acids, and vitamin C content can be better maintained in comparison with PE.	The developed films exhibited favorable mechanical properties at 20% RH (TS > 20 MPa and EAB > 15%).	Low oxygen and air barrier capacity due to phase separation when nisin was incorporated. Low mechanical performance at 90% RH.	[30]
**Category: Antioxidant, anti-browning and antibacterial active packaging**							
Chitosan (1.5% *w*/*v*)/polyethylene glycol (PEG 400) (50% *w*/*w*)	Anacardic acid (5, 10, 15 mg/100 mL)	The film-forming solution was dried using a conductive hydro drying setup.	Apple slices (3 × 1.7 × 1.5 cm) wrapped in films and stored for 6 h. No other storage conditions were reported.	The highest film antioxidant activity was 28.4% (DPPH assay) for 15 mg treatment. Furthermore, individually, chitosan and anacardic acid exhibited proven antimicrobial activity against *E. coli*, *L. monocytogenes*, *S. faecalis*, *S. thypi* and *S. boydii*. After 6 h, the films showed the capacity for browning inhibition (browning index near 4) and weight loss control (between 15% and 20%).	No significant changes were observed in the mechanical properties (TS 0.029 MPa, EAB 119%). Films biodegradability in soil was developed.	Despite the hydrophobicity of anacardic acid, films exhibited high water solubility. The biodegradability of chitosan film with Anacardic acid at 30 days was lower (62%) than that of chitosan film alone (70%) due to its high hydrophobicity	[61]
Pectin (3% *w*/*v*)/polyethylene glycol (PEG) (25% *w*/*w* pectin)	Grapefruit methanolic extract (0.04% *w*/*w*) and maltodextrin encapsulated lemon extract (0.04% *w*/*w*)	The film-forming solution was cast and spread into films with a film applicator.	Cherry tomatoes were cut in half, wrapped with active films, and stored in polystyrene boxes at 4 °C for 6 days.	Each citric-based material has antioxidant capabilities (pectin, grapefruit extract and lemon extract), which increase the total phenolic content of the film. The maximum activity is obtained when all three are present (84.73 DPPH). Incorporating citric extracts led to proven antibacterial activity against *S. aureus*, *S. enteriditis*, *E. coli*, and *K. pneumoniae*. Packaging artificially contaminated (with *E. coli*) cherry tomatoes and storing them at 6 °C demonstrated the antibacterial activity of the active films, as the original count population of 5.28 Log CFU/g decreased to 3.69 Log CFU/g on the 6th day.	The films have UV-light barrier capacity. Thermal stability (near 16% mass loss) in the temperature range of 200–250 °C. Flexibility over 10% EAB. Films biodegradability in soil within 28 days was 84%.	Low water solubility resistance due to abundant hydrophilic groups.	[50]
Chitosan (2% *w*/*v*)/Guar gum (no reported fraction)	Watermelon rind extract (1, 2, and 4%wt)	The film-forming solution was cast into films.	Banana (2 cm height slices) packaged in two 12 × 12 cm films and sealed into bags. Storage for 5 days; no other conditions reported.	Chitosan/guar gum films exhibited 20% DPPH scavenging activity. The watermelon rind extract increased the performance by up to 84%. Both chitosan and the natural extract provided the film with antibacterial activity, being effective against *E. coli* and *S. aureus* pathogens. By measuring the inhibition zone in agar, the inclusion of watermelon rind extract increased from 10 mm to nearly 16 mm. The inclusion of extracts improved the shelf-life performance of the fruit, as measured by color, firmness, weight loss, soluble solid content, and sensory evaluation. Additionally, the active packaging developed maintained the appearance of the fruit for two days more than the control polymer, from 2 to 4 days.	The materials exhibited thermal resistance (near 30% mass loss) up to 250 °C. Additionally, they developed UV-light barrier capacity and an improved tensile strength (>34.23 MPa)	Low elongation at break (below 5.5%) due to the presence of failure points and a disturbed matrix structure. Additionally, the water-vapor barrier should be more hydrophobic.	[51]
Soy protein isolate (SPI) (5%wt)/Carboxymethyl cellulose (CMC) (2%wt) Glycerol (3%wt)	Cinnamon and *Litsea cubeba* essential oils in polydopamine nanocapsules (EP) (0.125, 0.25, 0.5, and 1%wt)	The film-forming solution was cast into films.	Apple pieces (1 cm × 1 cm^2^) placed in sterile cups covered with the developed films and stored at 4 °C.	SPI-CMC film exhibited values of 2.4% in DPPH assays and 18.7% in ABTS assays. The addition of essential oil nanocapsules significantly improved the antioxidant activity to values near 66.6% and 98.6% in the DPPH and ABTS assays, respectively, for the 1%wt nanocapsule formulation. Antibacterial activity was developed due to essential oil release being effective against *E. coli* and *S. aureus* bacteria. The films delayed browning and the deterioration of cut apples, improving the shelf life from 12 h in control films to more than 24 h in 1%wt essential oil capsule films.	The thermal resistance of the materials was between 15% and 31% mass loss until 200 °C. A 0.5%wt EP film exhibits a better mechanical performance (TS: 8 MPa; EAB: 150%). One %wt exhibits a better hydrophobicity (107° CA). The EP films have 100% UV protection. And films biodegradability in soil was over 90% within 32 days.	The color changed to dark brown. The swelling ratio increased due to the high porosity of the films.	[12]
Methylcellulose (M) (2% *w*/*v*)/Glutaraldehyde (G) crosslinker (10 mL, 2%)	Noni leaf extract (20 mL, 0.005% *w*/*v*)	The film-forming solution was cast into films.	Apple syrup and apple slices were covered with the films and stored at room temperature for 120 h.	The inclusion of noni leaf extract increased the antibacterial activity from 11 mm in pristine films to over 13 mm against *S. aureus*, *B.* subtilis, *E. coli*, *P. aeruginosa* bacteria and *C. albicans* fungi. Furthermore, antioxidant performance was improved from 20.1% and 28.9% to 93.1% and 90.5% in the DPPH and ABTS assays, respectively. Browning, pH loss, and weight loss were effectively delayed for 120 h. In addition, the bioactivity of apples was significantly maintained, indicating their quality was appropriate after storage.	The tensile strength was improved (41.8 MPa) by the incorporation of Noni leaf extract. Furthermore, the hydrophobicity of the material increased (90.3° CA), enhancing its vapor barrier properties. MG and MGN films have degradability proved in soil burial test.	The elongation at break decreased slightly, caused by reduced chain mobility due to the incorporation of noni leaf extract. The biodegradability of MG films with Noni leaf extract at 30 days was lower (61.7%) than that of MG films (70.2%) due to possible soil bacteria actions inhibition.	[52]
**Category: Antioxidant-releasing active packaging**							
Cellulose acetate thermoplastic (CAT)	Potassium sorbate in layered double hydroxide (LDH-sorbate) (1.25, 2.5, and 5%wt)	The composite mixture was extruded into bags.	Pomegranate arils were stored in bags made from the developed films at 8 °C and 20 °C for 10 days.	The inclusion of sorbate in the films promoted a sustainable release of active particles. LDH–sorbate cellulose films released active substances after 120 days. A physiological deterioration in aril was effectively delayed and improved from 10% and 25% in Cat and polyethylene film packaging, respectively, to 5% deterioration in composite LDH-sorbate-CAT packaging after 10 days of storage.	Films have thermal resistance (30% mass loss) up to near 295 °C.	Up to 5%wt concentration the tensile strength decreased (20MPa). The elongation at break was significantly decreased (<9%) due to discontinuities in the polymer matrix.	[9]
Starch (6% *w*/*v*) Glycerol (2% *v*/*v*)	Liposome: Vitamin C capsules (0.1% *w*/*v*)	The film-forming solution of gelatinized starch/glycerol and VC capsules was cast into films.	Apple pieces (1 cm width × 2.5 cm diameter) were wrapped in the films and stored at different pHs (4 and 7) and temperatures (20, 25 and 37 °C)	The active film can release vitamin C into packaged fruit for 3 to 4 h. Low pHs and higher temperatures lead to faster release. Liposome encapsulation modified the diffusivity, which helped to control the release. Thus, the films exhibited prolonged activity.	Films have antioxidant capabilities provided by vitamin C.	No other information about film characteristics was reported.	[66]

--- Refers to the absence of fillers in the active packaging film developed in the study.

## Data Availability

Data are contained within the article.
